# Cigarette Smoke Extract Induces a Phenotypic Shift in Epithelial Cells; Involvement of HIF1α in Mesenchymal Transition

**DOI:** 10.1371/journal.pone.0107757

**Published:** 2014-10-16

**Authors:** Irene M. J. Eurlings, Niki L. Reynaert, Twan van den Beucken, Harry R. Gosker, C. C. de Theije, Fien M. Verhamme, Ken R. Bracke, Emiel F. M. Wouters, Mieke A. Dentener

**Affiliations:** 1 Department of Respiratory Medicine, NUTRIM School for Nutrition, Toxicology and Metabolism, Maastricht University Medical Center, Maastricht, the Netherlands; 2 Department of Radiation Oncology, GROW School for Oncology and Developmental Biology, Maastricht University Medical Center, Maastricht, the Netherlands; 3 Laboratory for Translational Research in Obstructive Pulmonary Diseases, Department of Respiratory Medicine, Ghent University Hospital, Ghent, Belgium; Helmholtz Zentrum München/Ludwig-Maximilians-University Munich, Germany

## Abstract

In COPD, matrix remodeling contributes to airflow limitation. Recent evidence suggests that next to fibroblasts, the process of epithelial-mesenchymal transition can contribute to matrix remodeling. CSE has been shown to induce EMT in lung epithelial cells, but the signaling mechanisms involved are largely unknown and subject of this study. EMT was assessed in A549 and BEAS2B cells stimulated with CSE by qPCR, Western blotting and immunofluorescence for epithelial and mesenchymal markers, as were collagen production, cell adhesion and barrier integrity as functional endpoints. Involvement of TGF-β and HIF1α signaling pathways were investigated. In addition, mouse models were used to examine the effects of CS on hypoxia signaling and of hypoxia per se on mesenchymal expression. CSE induced EMT characteristics in A549 and BEAS2B cells, evidenced by decreased expression of epithelial markers and a concomitant increase in mesenchymal marker expression after CSE exposure. Furthermore cells that underwent EMT showed increased production of collagen, decreased adhesion and disrupted barrier integrity. The induction of EMT was found to be independent of TGF-β signaling. On the contrary, CS was able to induce hypoxic signaling in A549 and BEAS2B cells as well as in mice lung tissue. Importantly, HIF1α knock-down prevented induction of mesenchymal markers, increased collagen production and decreased adhesion after CSE exposure, data that are in line with the observed induction of mesenchymal marker expression by hypoxia *in vitro* and *in vivo*. Together these data provide evidence that both bronchial and alveolar epithelial cells undergo a functional phenotypic shift in response to CSE exposure which can contribute to increased collagen deposition in COPD lungs. Moreover, HIF1α signaling appears to play an important role in this process.

## Introduction

Chronic obstructive pulmonary disease (COPD) is characterized by airflow limitation that is progressive and not fully reversible. The airflow limitation is often ascribed to remodeling, which consists of airway wall thickening and/or emphysema [1]. The association between remodeling and cigarette smoking, the main risk factor for COPD, has been established in both human and animal models [2], [3]. Most of these studies show airway wall thickening based on image analysis. Studies into the molecular changes in extracellular matrix associated with small airway remodeling in COPD and mechanisms involved are however few, and mainly focus on collagen, fibronectin and glycosaminoglycan deposition [4], [5]. In addition, some studies suggest that alveolar septae show signs of fibrosis by accumulation of collagen [6], proteoglycans [7], [8] and the glycosaminoglycan hyaluronan [9]. Although the bronchial and alveolar compartment are mostly considered independent entities we recently showed that the matrix of small airway walls and alveolar walls of COPD patients displays very similar features of remodelling [10].

In general fibroblasts are considered to be the main producers of extracellular matrix components [11]. Fibroblasts of COPD patients display enhanced expression of matrix proteins and release factors that promote further recruitment and activation of fibroblasts [12]. Interestingly, recently it was shown that the epithelium plays an important role in collagen deposition as well using an epithelial specific collagen 1 knock out mouse model [13]. The mechanistic process underlying the remodeled matrix as well as the role of activated epithelium in the remodeling process in COPD still needs to be further elucidated. One possible mechanism by which epithelium could contribute to enhanced matrix deposition is epithelial-mesenchymal transition (EMT), a process whereby epithelial functionality is repressed or lost and mesenchymal gene expression is attained. EMT has been shown to occur in interstitial pulmonary fibrosis by co-localisation of epithelial and mesenchymal proteins [14]. *In vitro* stimulation with transforming growth factor-β1 (TGF-β1) is mostly used to study the process, using enhanced expression of mesenchymal markers like desmin, collagen, vimentin, α-smooth muscle actin (α-SMA) and fibronectin, in concert with attenuated expression of epithelial specific genes like the adhesion molecule E-cadherin, cytokeratins, and tight junction proteins as a readout.

In COPD, the presence of EMT in airway wall biopsies was suggested, although this was only based on individual stainings of epithelial and mesenchymal markers by immunohistochemistry as opposed to double stainings [15]. Studies in H358, BEAS-2B, A549 and primary cells, furthermore showed the ability of human lung epithelial cells to undergo EMT *in vitro* following cigarette smoke extract (CSE) stimulation [16]–[18]. Most studies on EMT use *in vitro* stimulation with TGF-β [19], which has been implicated in remodeling and fibrosis. Reports on the role of TGF-β in COPD are however conflicting [20]–[26], and the role of TGFb signaling in CSE-induced EMT in vitro is not elucidated. Another mechanism that could underlie EMT in response to CSE is hypoxia signaling as hypoxia was shown to induce EMT in A549 cells, which was HIF1α dependent [27]. Importantly, HIF1α was found to be increased in lung tissue of COPD patients in relation to structural changes of the bronchial epithelium and subepithelial fibrosis [28].

Therefore, the aim of this study was to investigate signaling mechanisms involved in CSE-induced EMT in epithelial cells. As we previously showed matrix remodeling in both alveolar and bronchial walls of COPD patients [10], both A549 and BEAS2B cells were used in this study. Extensive investigation did not show contribution for TGF-β signaling in the induction of EMT by CSE. On the other hand HIF1α was documented to be essential to the induction of a mesenchymal phenotype in response to CSE, and hypoxia per se mimicked these effects *in vitro* and *in vivo*.

## Methods

### Cell culture

A549 and BEAS2B and H292 cell lines (ATCC, Manassa, VA, USA) were cultured as described previously [29], [30]. At>90% confluency, cells were starved and stimulated, twice with CSE every 24 h.

For air liquid interface cell cultures, A549 cells were plated on 0.4 µM pore size transwells (Corning, NY, USA) and cultured until confluency. Apical medium was removed and after 72 h medium in the bottom well was replaced by starvation medium. Cells were stimulated by addition of CSE at the apical surface for 2 h after which it was removed. This procedure was repeated on each of the next two consecutive days.

### Cell viability

Medium was collected and adhering cells were trypsinised at indicated time points. A 1∶2 dilution of the cells in Trypan Blue (T8154; Sigma-Aldrich) was made and transferred onto a cover-slipped haemocytometer. Using phase-contrast light microscopy, viable cells were identified as rounded and bright, whereas blue cells were considered nonviable. A cell count and the calculation of percentage viability were recorded.

### Fluorescein leakage test

Fluorescein leakage test was performed as previously described [31]. Hanks' balanced salt solution (HBSS) was used throughout as washing buffer and as vehicle for Na-fluorescein. After culturing A549 cells ALI, cells were then washed with HBSS and the inserts were placed in fresh plates containing HBSS. Na-fluorescein (500 µl, 10 pg/ml) was then added to and the apical site and incubated for 2 hours. The inserts were removed and the amount of fluorescein in each well was determined basolateral at an excitation wavelength of 485 nm and an emission wavelength of 530 nm.

### Adhesion assay

After stimulation with CSE for 48 hours in tissue culture flasks, cells (1*10∧5 per well) were seeded into 24-wells plates wells and incubated for two hours at 37°C in a 5% CO2 incubator. Non-adherent cells were removed and the wells were washed twice with PBS. The extent of cell adhesion was determined after fixation of the adherent cells with 4% PFA for 10 minutes and drying overnight at room temperature. Cells were stained for 20 minutes at room temperature with 0.1% crystal violet to stain the cells, washed with water and dried. Stained cells were solubilized in 10% SDS, and absorbance at 750 nm was determined.

### Exposure of mice to chronic hypoxia

Mice were treated as described previously [32]. In short, 52 week (wk) old C57BL/6J mice (Charles River Laboratories, Wilmington, MA) were exposed to ambient air (normoxia, n = 8) or chronic hypoxia (n = 7) for 21 days. To control for the effects of reduced food intake during hypoxia, another group of mice was exposed to ambient air while receiving the amount of food consumed by the hypoxic mice (pair-fed group, n = 8). All mice were housed in experimental chambers at 21°C with a 12-h dark/light cycle. Mice received standard chow (V1534–000 ssniff R/M-H, ssniff Spezialdiäten, Soest, Germany) and water ad libitum. Using the proOX P110 (BioSpherix, Lacona, NY) system, O2 was replaced by N2 in a stepwise manner to create normobaric oxygen levels of 12% (day 1), 10% (day 2), and finally 8% (60.8 mmHg) on day 3. The latter oxygen concentration was maintained until day 21. Three to four mice were housed per cage. Daily food intake was determined per cage, and mice were weighed daily. On day 21, mice were sacrificed. The lungs were snap frozen in liquid nitrogen and stored immediately in −80°C. The protocol was approved by the Committee for Animal Care and Use of Maastricht University (project 2009-151).

### Smoking mouse model

Male C57BL/6 mice, 6 to 8 weeks old, were purchased from The Jackson Laboratory (Bar Harbor, ME, USA). The local Ethics Committee for animal experimentation of the faculty of Medicine and Health Sciences (Ghent University, Belgium) approved all *in vivo* manipulations.

Mice (n = 5 per group) were exposed whole body to CS as described previously [33]. Briefly, groups of five mice were exposed to the tobacco smoke of five cigarettes (Reference Cigarette 3R4F without filter; University of Kentucky, Lexington, KY, USA) four times a day with 30-minute smoke-free intervals, 5 days per week for 24 weeks. An optimal smoke/air ratio of 1∶6 was obtained. The control group was exposed to air. 24 hours after the last exposure, mice were sacrificed by an intraperitoneal injection of pentobarbital (CEVA-Sanofi, Paris, France).

### Cigarette smoke extract

Filters were removed from 3R4F Research cigarettes (University of Kentucky) and CSE was made using a linear pump which bubbled air (2 mL/sec) through 2 mL HBSS (Gibco) for 5 seconds followed by a pause for 5 seconds according to Carp et al. [34]. Cells were stimulated within 15 minutes with 2.5% CSE for A549 cells and 1% for BEAS2B cells.

### HIF1a knock-down

Lentiviral particles, generated with or without the shRNA construct TRCN0000003810 were used to achieve stable knock-down of HIF1α. A549 and BEAS2B cells as described previously [35].

### Luciferase reporter assay

Transient transfections were performed using X-tremeGENE HP (Roche, Penzburg, Germany) according to the manufacturer's instruction using 0.875 µg SMAD [36] or Hypoxia-responsive element (HRE) [37] promotor luciferase plasmids and 0.125 µg pSV-β-galactosidase to correct for transfection efficiency. Luciferase (Promega) and β-galactosidase (Tropix) activities were measured according to the manufacturers' instructions.

### qPCR

RNA was isolated from cells using the High Pure RNA Isolation Kit (Roche) or mouse lung tissue using the RNeasy kit (Qiagen, CA USA) and reverse transcribed using Transcriptor First Strand cDNA Synthesis Kit (Roche). PCR reactions were performed on an ABI 7900HT apparatus (Applied biosystems, Foster City, CA, USA) using SYBR green dye (Applied biosystems) and primer sequences are listed in table 1.

**Table 1:** Primer sequences.

Gene	Forward primer 5′ to 3′	Reverse primer 5′ to 3′
Human		
E-cadherin	TCATGAGTGTCCCCCGGTAT	GTCAGTATCAGCCGCTTCAGAT
Keratin 7	GGGACTGCAGCTCTGTCAAC	CTGCCTACATGAGCAAGGTG
Keratin 18	CGGGCATTGTCCACAGTATT	GGGAGCACTTGGAGAAGAAG
α Smooth muscle actin	GAGAAGAGTTACGAGTTGCCTGA	TGTTAGCATAGAGGTCCTTCCTG
Plasminogen activator inhibitor 1	GAAAGTGAAGATCGAGGTGAACGAGA	CATGCGGGCTGAGACTATGACA
Vimentin	ATTCCACTTTGCGTTCAAGG	CTTCAGAGAGAGGAAGCCGA
Transforming growth factor-β	TGAACCGGCCTTTCCTGCTTCTCATG	GCGGAAGTCAATGTACAGCTGCCGC
SMAD7	TACCGTGCAGATCAGCTTTG	TTTGCATGAAAAGCAGCAC
Carbonic anhydrase 9	CATCCTAGCCCTGGTTTTTGG	GCTCACACCCCCTTTGGTT
Ribosomal Protein L13a	CCTGGAGGAGAAGAGGAAAGAGA	TTGAGGACCTCTGTGTATTTGTCAA
Mouse		
Plasminogen activator inhibitor 1	AGTCTTTCCGACCAAGAGCA	GACAAAGGCTGTGTGGAGGAAG
Fibronectin	GTGTAGCACAACTTCCAATTACGAA	GGAATTTCCGCCTCGAGTCT
Ribosomal protein L13a	CACTCTGGAGGAGAAACGGAAGG	GCAGGCATGAGGCAAACAGTC
Carbonic anhydrase 9	CAGGAGGCCTGGCAGTTTT	TTCTTCCAAATGGGACAGCAA

### Western blotting

Protein lysate, obtained by cell lysis using RIPA buffer (150 mM NaCl, 1% Nonidet, 0.5% NA deoxychholate, 0.1% SDS in 50 mM Tris), was separated on 12% Criterion XT Bis-Tris gels (Bio-Rad, Hercules, CA, USA) and transferred onto a nitrocellulose membrane (Protran, Kent, UK). For all Western blots 20 ug protein lysate was used, except for the detection of vimentin in BEAS2B cells for which 2 µg protein was sufficient. Blocking in 5% milk was followed by primary antibody against E-cadherin (#3195, 1/2000 dilution), ZO-1 (#8193, 1/1000 dilution), vimentin (#5741, 1/5000 dilution) and GAPDH (#2118, 1/10000 dilution)(all Cell Signaling, Danvers, MA, USA) and peroxidase-conjugated secondary antibody (Vector), which was detected with SuperSignal West Pico Chemiluminescent Substrate (Thermo Fisher, Waltham, MA, USA).

### Immunocytochemistry

Cells grown on slides (Thermo Scientific, Waltham, MA, USA) were treated as indicated and fixed with 4% paraformaldehyde, permeabilized using PBS/0.1% Triton X-100, and blocked with 5% BSA. Primary antibodies (1/100) against SNAIL (#3879) and ZO-1 (#8193) (Cell Signaling) were used in conjunction with Alexa 555labeled anti-rabbit IgG conjugates (Molecular Probes, Eugene, OR), and 5 µg/mL DAPI. Fluorescence was visualized on a Nikon eclipse E800 (Nikon, Melville, NY, USA) at 20 times magnification.

### Collagen assay

Collagen content of cell free medium was determined by the addition of Sirius Red staining reagent (Klinipath, Duiven, Netherlands) and 0.5% Triton X-100 (Sigma) in saturated Picric acid (Klinipath)). The precipitate obtained after centrifugation was dissolved in 0.5M NaOH. Extinction was measured at 540 nm and concentrations were calculated using a standard curve.

### Statistical analyses

Between-group comparisons were analyzed using the Kruskal-Wallis test, followed by the Mann-Whitney U test (SPSS20). Data were expressed as mean and standard deviation. A p-value<0.05 was considered statistically significant.

## Results

### CSE stimulation decreased epithelial markers in lung epithelial cells

**Figure 1:**
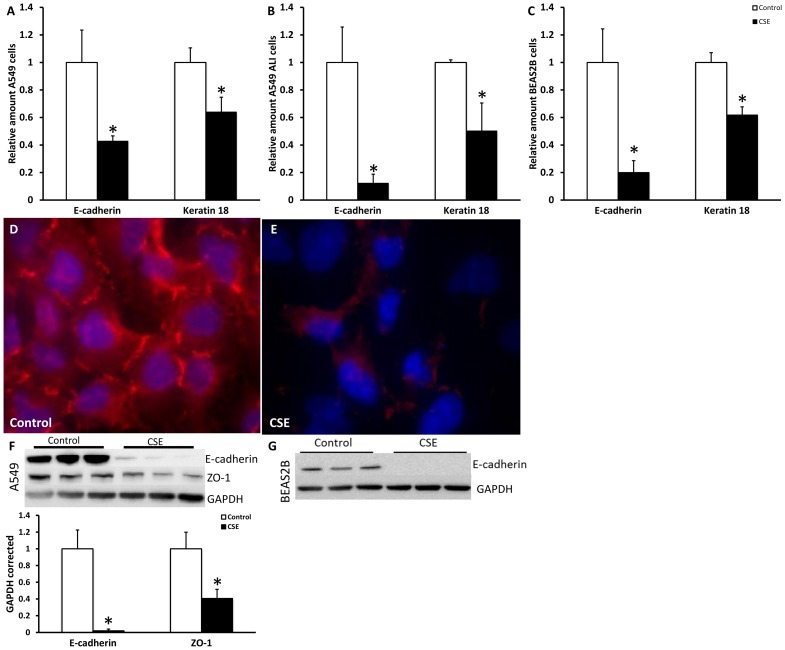
CSE decreased epithelial markers in lung epithelial cells. Cells were treated for 48 hours with respectively 2.5% CSE for A549 cells and 1.0% CSE for BEAS2B cells. mRNA levels of E-cadherin and Keratin 18 were measured in A549 cell submerged (A), A549 cells at ALI (B) and BEAS2B cells (C) via qPCR. Data are expressed as mean+SD, * indicates *p*<0.05 compared to untreated controls. ZO-1 staining (red) of untreated A549 cells (D) and A549 cells treated for 48 hours with CSE (E) detected by immunofluorescence. Nuclei are counterstained using DAPI. E-cadherin and ZO-1 Western blots and quantification of untreated controls and A549 cells treated for 48 hours with 2.5% CSE (F) and BEAS2B with 1.0% CSE (G) in triplicate using GAPDH as a loading control.

EMT involves the repression of epithelial markers such as the adhesion molecule E-cadherin, the tight-junction protein ZO-1 and keratins. To test the effect of cigarette smoke on these markers we first tested the effect of CSE on viability of A549 and BEAS2B cells. Results in figure S1 showed that a dose of 2.5%CSE for A549 and 1.0% for BEAS2B cells did not cause significant cell death or changes in number of cells. After stimulation for 48 hours with these concentrations, mRNA levels of E-cadherin and keratin 18 were significantly decreased due to CSE treatment in cultures of A549, BEAS2B (Fig. 1) and H292 cells (data not shown). Intriguingly, effects of CSE were even more pronounced in ALI compared to submerged cultures of A549 (Fig. 1B). Furthermore, at the protein level reduced amounts of ZO-1 by immunofluorescence, and E-cadherin and ZO-1 by Western blotting were found after 48 hours of CSE treatment compared to untreated A549 cells (Fig. 1D–F). In BEAS2B cells E-cadherin protein expression was clearly present in untreated cells and completely lost after CSE treatment (Fig. 1G), therefore no quantification was performed. Together these data indicate that CSE stimulation leads to an attenuation of the epithelial phenotype of multiple lung epithelial cells.

### Mesenchymal phenotype is increased after CSE stimulation

**Figure 2:**
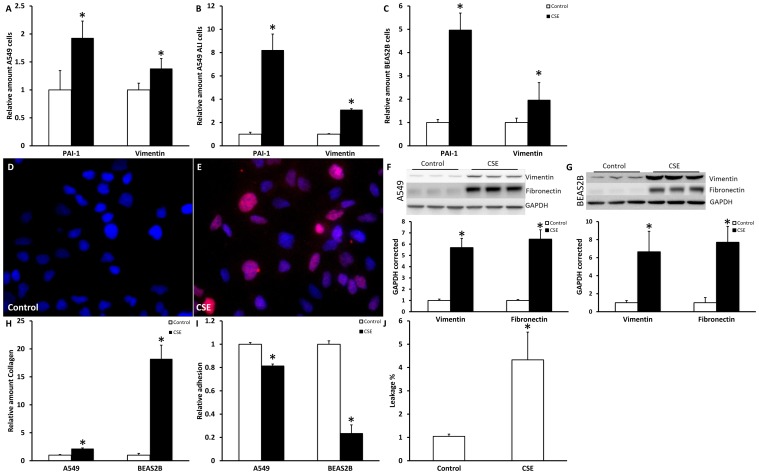
CSE increased mesenchymal markers in lung epithelial cells. Cells were treated for 48 hours with respectively 2.5% CSE for A549 cells and 1.0% CSE for BEAS2B cells. mRNA levels of PAI-1 and vimentin were measured in A549 cell submerged (A), A549 cells at ALI (B) and BEAS2B cells (C) via qPCR. Data are expressed as mean+SD, * indicates p<0.05 compared to untreated controls. SNAIL staining of untreated control A549 cells (D) and A549 cells stimulated with CSE for 48 hours (E) as detected by immunofluorescence (red). Nuclei are counterstained using DAPI. Vimentin and fibronectin Western blot and quantification of untreated controls and 48 hours CSE treated A549 cells (F) and BEAS2B cells (G) in triplicate using GAPDH as a loading control. Collagen in the medium, measured using sircoll assay (H), Adhesion of A549 and BEAS2B cells (I) and leakage of A549 cells grown ALI after CSE exposure (J).

To address whether the observed attenuation of epithelial markers coincides with increased expression of mesenchymal markers, mRNA and protein levels of several typical mesenchymal markers were analyzed at the same time point. Data in figure 2 demonstrate that mRNA levels of PAI1 and vimentin were significantly increased after 48 hours of CSE stimulation in submerged (Fig. 2A) as well as ALI (Fig. 2B) cultures of A549 cells, BEAS2B cells (Fig. 2C) and H292 cells (data not shown). In line with data in Fig. 1, effects of CSE were again strikingly more pronounced in ALI cultures of A549 cells compared to submerged cell cultures. In addition protein levels of SNAIL, an EMT related transcription factor which represses E-cadherin, were increased in CSE treated A549 cells compared to untreated cells (fig. 2D–E). Also vimentin and fibronectin protein levels were increased after 48 hours of stimulation in A549 and BEAS2B cells as shown by Western blotting (fig. 2F–G). Taken together, these data in combination with the decrease in epithelial markers indicate that CSE induces EMT in various lung epithelial cells.

To check whether these epithelial cells in which EMT is induced by CSE underwent functional changes, collagen production in the medium was measured with the sircoll assay. As shown in figure 2H, CSE increased the production of collagen significantly in A549 as well as in BEAS2B cells. These data indicate that epithelial cells produce more collagen in response to CSE exposure. In addition, adhesion and barrier integrity were investigated as functional endpoints. Adhesion on cell culture plates was significantly decreased in A549 and BEAS2B cells after CSE exposure (Figure 2I) and similar results were obtained for adhesion on collagen I or fibronectin coated dishes (data not shown). We show that in ALI cultures of A549 no fluid added to the apical compartment was leaking to the basolateral site, indicating a functional barrier (Figure S3A) and that, barrier integrity was partly disrupted after CSE exposure (Figure 2J).

### CSE-induced EMT in A549 cells is not mediated via TGF-β or the MAPK signaling pathway

**Figure 3:**
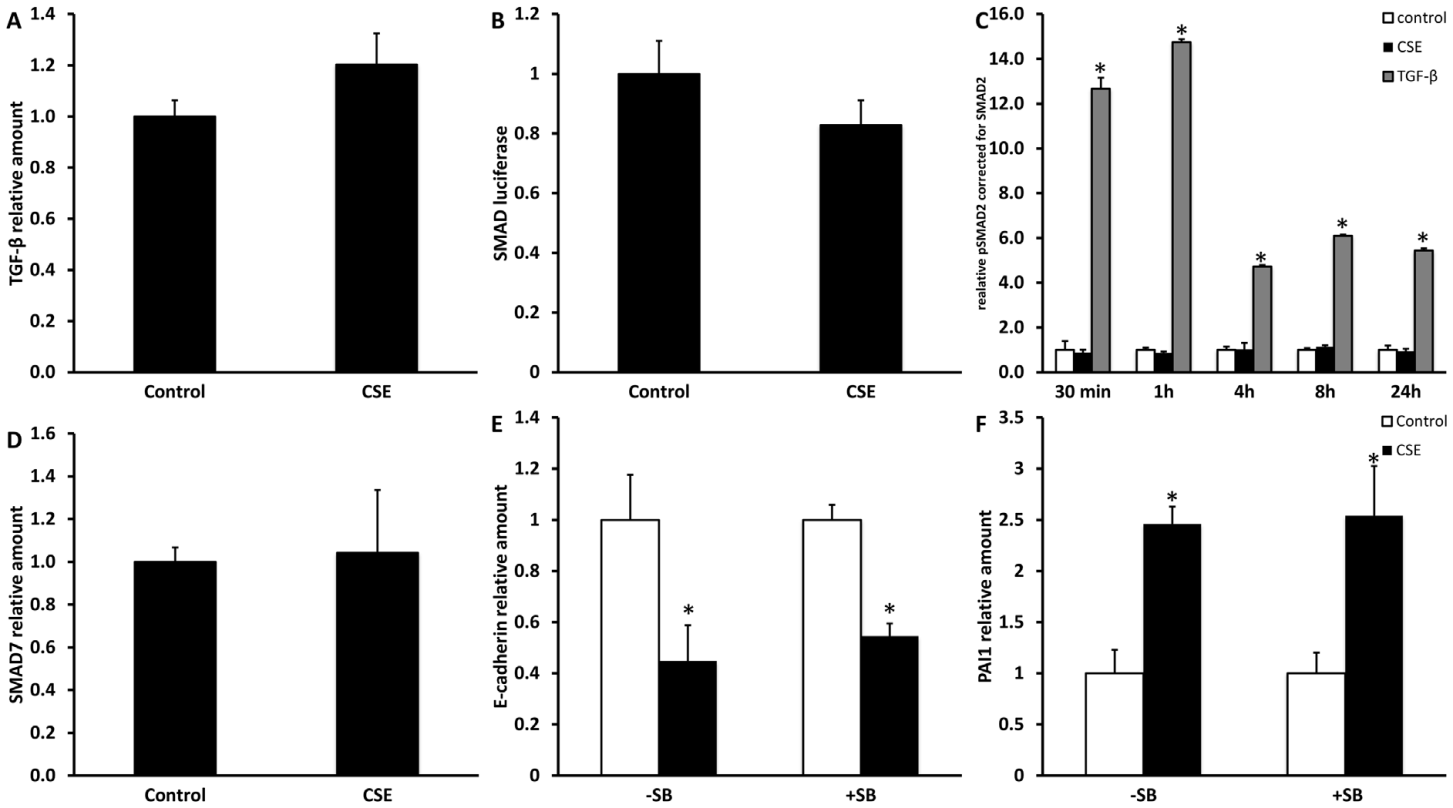
The TGF-β signaling pathway is not involved in CSE-induced EMT. Submerged grown A549 cells were treated for 24 h with 2.5% CSE. Levels of TGF-β mRNA (A), SMAD reporter luciferase activity corrected for levels of β-galactosidase activity (B), SMAD2 phosphorylation as analyzed and corrected for total SMAD2 by Western blot (after stimulation with 2.5% CSE or 10 ng/mL TGF-β) (C) and SMAD7 mRNA (D) are shown. Effect of inhibition of the TGF-β signaling was studied using the receptor blocker SB431542 which was pre-incubated at a dose of 10 µM for 30 minutes followed by CSE stimulation for 48 hours. E-cadherin (E) and PAI1 (F) mRNA levels were measured by qPCR. Data are expressed as mean+SD, * indicates p<0.05 between CSE stimulated and untreated control.

TGF-β, a multifunctional cytokine that regulates tissue morphogenesis and differentiation through effects on cell proliferation, differentiation, apoptosis and ECM production, has been identified as a main player in EMT. To investigate whether CSE-induced changes in expression of epithelial and mesenchymal markers observed in A549 cells occurred via TGF-β, several approaches were used. First, it was investigated if CSE stimulation leads to increased TGF-β expression. No changes were however found at the mRNA (Fig. 3A) or protein (data not shown) level. Next, no significant increases in SMAD luciferase reporter activity and phosphorylation of SMAD2 were found in response to CSE stimulation (Fig. 3B–C), whereas clear inductions were observed when cells were stimulated with TGF-β.

Although no effect of CSE on the stimulatory SMAD signaling pathway was observed, CSE could induce EMT via effects on the inhibitory SMAD pathway. Results in Fig. 3D show that also mRNA levels of inhibitory SMAD7 were unaffected by CSE exposure. Lastly, the TGF-β pathway was blocked by using SB431542. Blocking of the phosphorylation domain on the TGF-β receptor by pre-treatment with this SB compound did not rescue the decreased E-cadherin expression nor did it prevent the increased expression of PAI 1 by CSE (Fig. 3E–F). As control for SB activity, its effect on TGF-β induced EMT, as investigated by expression of E-cadherin and PAI1, was analyzed which was almost completely blocked (Fig. S2).

Next to the classical TGF-β signaling pathway, we examined the involvement of the MAPK signaling pathway, by inhibition of Erk1/2 using pre-incubation with the inhibitor U0126. Erk inhibition had no effects on CSE-induced EMT, whereas TGF-β induced EMT was prevented by the use of U0126 (Fig. S2).

### Hypoxia signaling is induced by CSE *in vitro* and *in vivo*

**Figure 4:**
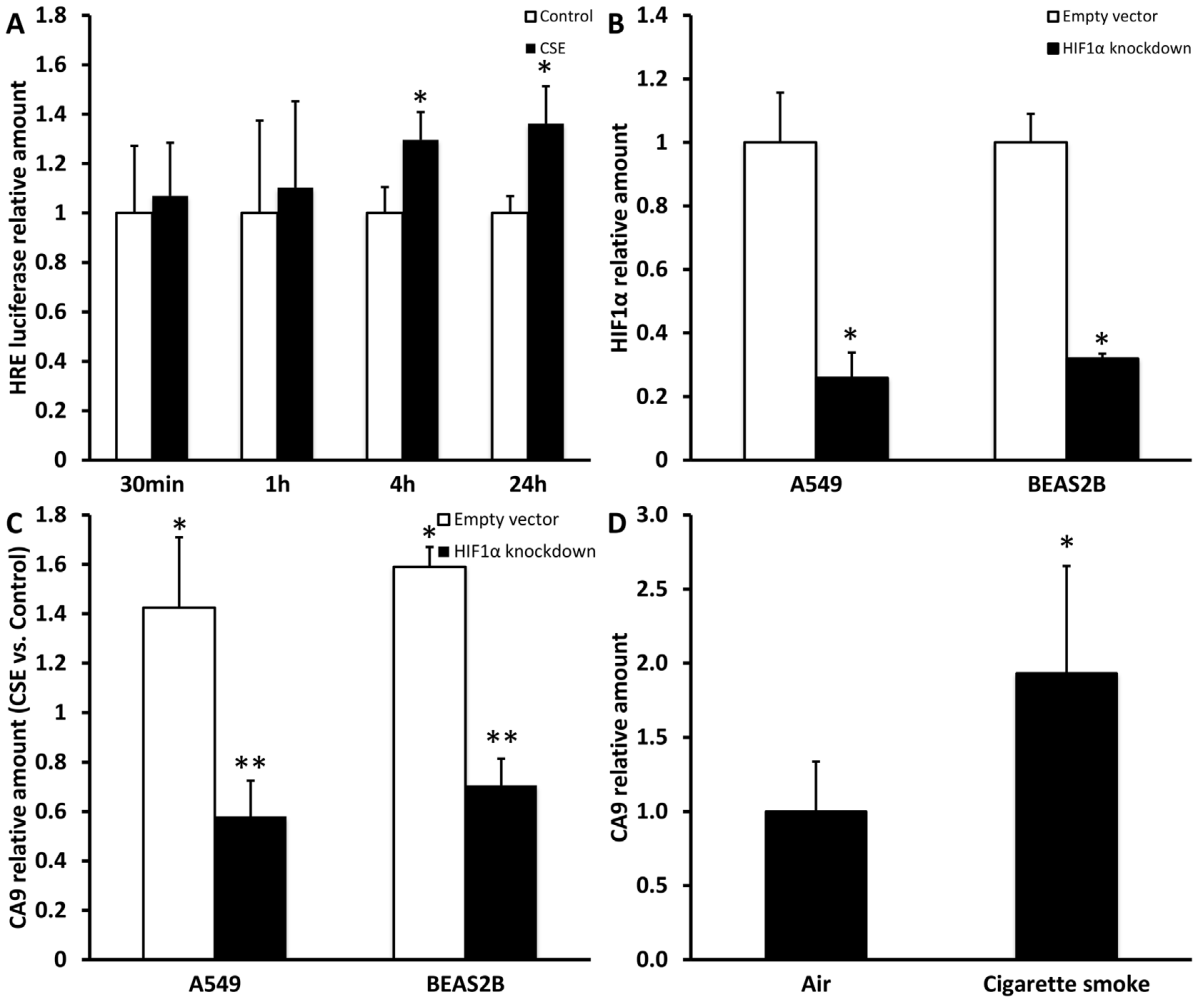
CSE induces hypoxia signaling. A549 and cells were treated for several time points with 2.5% CSE. (A) Levels of HRE reporter luciferase corrected for levels of β-galactosidase activity. Relative HIF1α mRNA expression in A549 and BEAS2B cells with stable HIF1α knock-down or PLKO.1 empty vector (B). Relative CA9 mRNA expression after 24 h of CSE stimulation compared to untreated control in A549 and BEAS2B cells with stable HIF1α knock-down or PLKO.1 empty vector(C). (D) CA9 mRNA expression in lung tissue of mice exposed to air or cigarette smoke for 6 months. Data are expressed as mean+SD, * indicates p<0.05 compared to control, ** indicates p<0.05 compared to empty vector.

In a rat model for COPD, using exposure to LPS and cigarette smoke it was shown that expression of hypoxia inducible factor (HIF) 1α responsive genes was increased [38] and hypoxia is furthermore reported to be able to induce EMT *in vitro* in alveolar epithelial cells [27]. To study induction of hypoxia signaling in alveolar epithelial cells by CSE, A549 cells were transfected with a hypoxia responsive element (HRE) luciferase reporter and exposed to CSE for several time points. Figure 4A shows a significant increase in luciferase activity at 4 and 24 h of stimulation. Furthermore, mRNA expression of the HIF1α responsive gene carbonic anhydrase 9 (CA9) was studied in A549 and BEAS2B cells in which HIF1α was stably down regulated using shRNA as shown in figure 4B. In line with the data in figure 4A, CA9 mRNA expression was significantly increased after treatment with CSE. This increase could be prevented and even inversed by the knock-down of HIF1α in both cell lines (Fig. 4C). In addition, in lung tissue of mice exposed to cigarette smoke for 6 months a clear and significant induction of CA9 mRNA expression was also present (Fig. 4D). Together, these data indicate that CSE induces hypoxia signaling via HIF1α in epithelial cells.

### Hypoxia induces mesenchymal markers *in vitro* and *in vivo*

**Figure 5:**
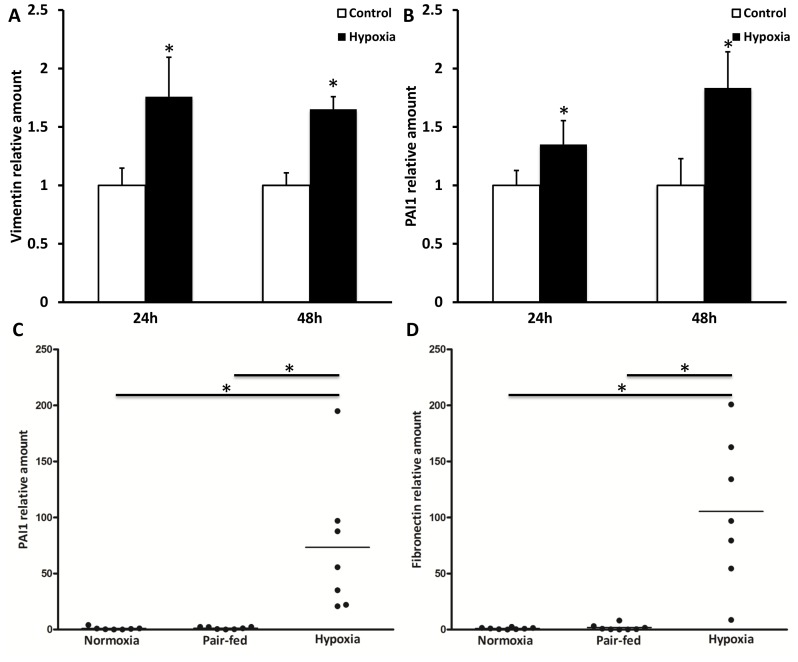
Hypoxia induces mesenchymal markers. A549 cells were cultured under normoxic or hypoxic conditions (4% O_2_) for 24 or 48 hours. Vimentin (A) and PAI1 (B) mRNA levels were measured by qPCR and are expressed as mean+SD, * indicates p<0.05 compared to control. C57BL/6J mice were exposed to normoxia (n = 8) or hypoxia (n = 7) for 21 days and additionally a pair-fed group (n = 8) was used. Lung fibronectin (C) and PAI1 (D) mRNA levels were determined by qPCR. Data are expressed as mean, * indicates p<0.05.

After demonstrating that CSE exposure induced HIF1α signaling in A549 cells, we assessed whether hypoxia could induce features of EMT *in vitro* in alveolar epithelial cells or *in vivo* in hypoxia exposed mice. No alterations in mRNA expression of epithelial markers E-cadherin, ZO-1 and keratin 7 and 18 were observed in A549 cells after 24 and 48 h of exposure to hypoxia or in lungs of mice exposed to hypoxia for 21 days (data not shown). On the other hand expression of mesenchymal markers was found to be increased due to hypoxia. In alveolar epithelial cells which were exposed to 4% O_2_ for 24 h or 48 h, a significant increase in vimentin and PAI1 mRNA levels was observed (Fig. 5A–B). In mice exposed to hypoxic conditions for 21 days mRNA expression of fibronectin and PAI1 was increased in total lung tissue compared to both normoxia and pair-fed treated animals (Fig. 5C–D).

### CSE induction of mesenchymal phenotype is HIF1α dependent

**Figure 6:**
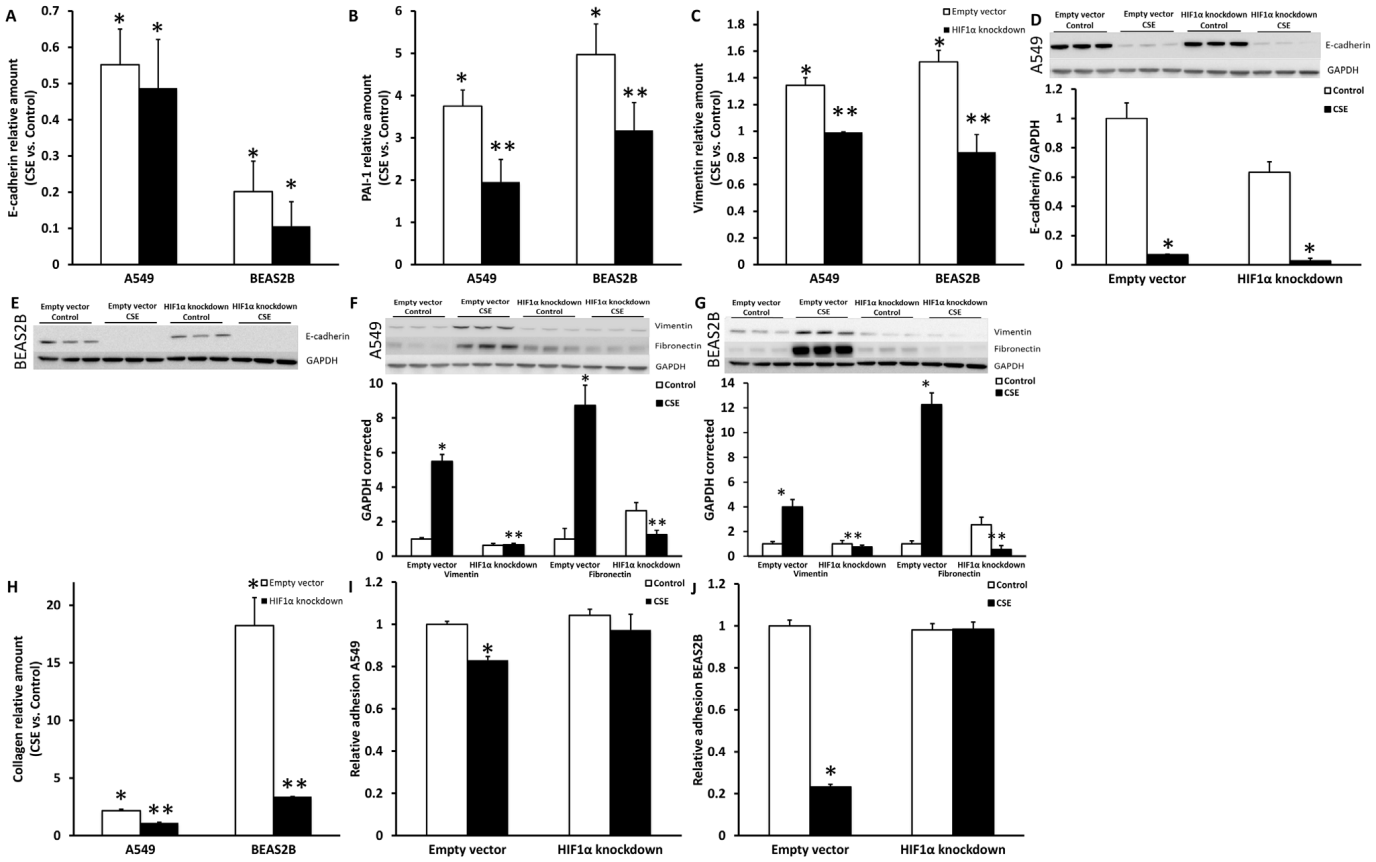
Induction of mesenchymal markers by CSE is HIF1α dependent. Relative E-cadherin (A), PAI1 (B) and vimentin (C) mRNA levels after 48 hours of CSE stimulation (A549 2.5% CSE and BEAS2B 1.0% CSE) compared to control in A549 and BEAS2B cells stably transfected with PLKO.1 empty vector and shRNA HIF1α construct. Western blots and quantification of A549 en BEAS2B cells stably transfected with PLKO.1 empty vector and shRNA HIF1α construct after stimulation with for 48 hours for E-cadherin (D–E), vimentin and fibronectin (F–G) with GAPDH as loading control. Collagen in the medium, measured using sircoll assay (H). Adhesion in A549(I) and BEAS2B cells (J). Data are expressed as mean+SD, * indicates p<0.05 compared to control, ** indicates p<0.05 compared to empty vector.

To examine whether the HIF1α signaling shown in figure 4 is involved in the CSE induced changes in expression of epithelial and mesenchymal markers, A549 and BEAS2B cells were stably transfected with a HIF1α shRNA construct. HIF1α knock-down did not change basal expression of the epithelial marker E-cadherin and could not prevent the decrease in E-cadherin mRNA or protein expression observed after CSE exposure (Fig. 6A and D–E). For, keratin 7 and 18 mRNA expression, similar data were obtained (data not shown). These results are in line with the lack of reduced epithelial marker expression under hypoxic conditions. Interestingly, HIF1α knock-down was able to prevent the induction of the mesenchymal markers PAI1, vimentin and fibronectin observed after CSE exposure in control cells at the mRNA (Fig. 6B–C) and protein level (Fig. 6. F–G), which is in agreement with effects of hypoxia per se shown in figure 5.

Next it was examined whether the prevented increase in mesenchymal markers by HIF1α knock-down also prevented the production of collagen by A549 and BEAS2B cells. Therefore collagen content was measured in the medium of empty vector and shHIF1α transfected cells after CSE stimulation. As shown in figure 6H, collagen production was significantly increased by CSE in the empty vector transduced cells. However in HIF1α knock-down cells, not only the basal levels of collagen production were decreased but also the CSE-induced collagen production was almost completely blunted in both cell lines. Together this indicates that CSE induces collagen production in epithelial cells via HIF1α signaling. Secondly, the decreased adhesion after CSE exposure in both A549 and BEAS2B cells was prevented completely by the knockdown of HIF1α (Figure 6I–J). These data indicate the involvement of HIF1a in the functional shift towards mesenchymal phenotype.

## Discussion

In this study it is shown that CSE induced EMT in alveolar and bronchial epithelial cells. Extensive investigation showed that there was no involvement for TGF-β induced SMAD or MAPK signaling. CSE was found to activate hypoxia signaling and hypoxia was found to induce a mesenchymal phenotype as was observed by the up regulation of mesenchymal markers in lung tissue of mice and in alveolar epithelial cells. Furthermore hypoxia signaling was found to play an important role in the upregulation of a mesenchymal phenotype as knock-down of HIF1α blunted enhanced expression of mesenchymal markers and interestingly also the production of collagen in response to cigarette smoke. In conclusion it was shown that HIF1α is involved in CSE-induced mesenchymal marker expression.

EMT is characterized by the decrease or loss of cobblestone morphology and a reduction in epithelial specific expression. The decrease in E-cadherin and Keratin 18 mRNA levels and furthermore the E-cadherin and ZO-1 protein levels in response to CSE observed in epithelial cells in this study is in line with previous results on epithelial marker loss in CSE stimulated primary bronchial epithelial cells [15]. Concurrently with the decrease of epithelial cell markers, A549 and BEAS2B cells gain a mesenchymal phenotype with increased expression of vimentin, PAI1 and SNAIL in response to CSE treatment, evidencing EMT. In addition to the gain of these classical mesenchymal markers, CSE stimulated cells showed decreased adhesion and increased leakage indicating a functional shift towards a mesenchymal phenotype. These data are supported by recent publications showing the loss of epithelial integrity by CSE [39] and the disruption of tight junctions and barrier function of bronchial epithelial cells [40]. The increased release of collagen into the medium due to CSE exposure could underlie the increased collagen content in both alveolar and small airway walls of COPD patients as we recently published [6], [10]. Together with the data in the epithelial specific collagen1 knock-out mouse showing limitation of fibrosis after bleomycin challenge [13], these data indicate that epithelial cells through a phenotypic switch are indeed a possible source of accumulation of ECM.

The process of EMT has been suggested to occur in several fibrotic lung disorders and contribute to enhanced matrix deposition. Providing proof of the occurrence of EMT *in vivo* remains however difficult, as the epithelial source of fibroblasts arising from “complete EMT” in clinical samples cannot be determined [41]. Furthermore, lineage tracing in transgenic mouse models provides controversial evidence for the occurrence of EMT in animal models. Despite these difficulties, “incomplete EMT” has been demonstrated in COPD patients by localization of the mesenchymal marker FSP1 in bronchial epithelial cells which were identified by cytokeratin staining [42].

As TGF-β has been shown to play an important role in EMT induction, we investigated the involvement of this signaling pathway in the observed CSE-induced EMT. Extensive examination of TGF-β expression, TGF-β signaling intermediates and blockage of the TGF-β receptor using a pharmacological inhibitor or inhibition of Erk1/2 did not provide any evidence of activation of this pathway by CSE or a role thereof in the induction of EMT by CSE. These findings are in contrast with data in primary human bronchial epithelial cells, which show increased levels of TGF-β as well as increased SMAD and Erk signaling after treatment with CSE [15]. Interestingly, literature on the role of TGF-β in COPD is conflicting as well. Some studies observed no differences in TGF-β levels in sputum, BALF and bronchial epithelial cells by immunostaining (27–31), whereas others found increased expression of TGF-β in bronchial and alveolar epithelial cells (25, 26).

In alveolar epithelium it was shown that hypoxia induced αSMA and vimentin where E-cadherin expression was decreased (12). Consistent with these findings, in the current study it was shown that hypoxia could induce mesenchymal marker expression in A549 cells as well as in lung tissue of hypoxia exposed mice. During hypoxia, HIF-1α protein stabilizes and forms a heterodimeric transcription factor with HIF-1β subunits [43], leading to transcription of genes participating in the cellular adaptation to hypoxia. Although in this study, cells were cultured under normoxic conditions, CSE was able to induce hypoxic signaling. Evidence for induction of hypoxic signaling by cigarette smoke *in vivo* was also found as lung tissue of mice exposed to cigarette smoke for 6 months displayed enhanced expression of CA9. Previous research from our group showed increased collagen and fibronectin content in the lungs of these mice [44], however the role of HIF1α in this should be further elucidated in future studies. One possible explanation is that CSE leads to an upregulation of HIF1α transcriptionally or that CSE stabilizes HIF1α as shown in response to nicotine under normoxic conditions [45]. Furthermore, it has been shown recently that HIF1α expression is induced by treatment of BEAS2B cells with side stream smoke [46].

Although others have shown EMT in response to hypoxia in A549 and other alveolar epithelial cells [27], we only found evidence for the induction of mesenchymal markers but not an attenuation of epithelial markers *in vitro* as well as *in vivo*. In line with these findings, decreased expression of epithelial cell markers in response to CSE could not be rescued by HIF1α signaling abrogation. This suggests that the loss of epithelial markers thus involves other signaling pathways than HIF1α or TGF-β. A possible mechanism for the decrease in epithelial markers could be a direct effect of CSE on HDAC recruitment and/or activation to the promotor of epithelial genes as was shown for E-cadherin [47].

On the other hand, in the current study data indicates that HIF1α signaling is necessary for induction of mesenchymal marker expression and increased collagen production in response to CSE. Importantly, also basal production of collagen was attenuated by HIF1α knock-down. In COPD a link between HIF1α and matrix remodeling was demonstrated by a study in which the number of HIF1α-positive epithelial cells in bronchial biopsies of COPD patients was shown to increase with reticular basement membrane thickness as a marker of sub epithelial fibrosis [28]. Together these data provide evidence that bronchial and alveolar epithelial cells undergoing a phenotypic shift in response to CSE can contribute to increased collagen deposition in COPD, in which HIF1α signaling plays an important role.

## Supporting Information

Figure S1 **No significant cell death is present after CSE exposure.** Cell death was measured using trypan blue staining in A549 and BEAS2B cells after stimulation for 48 h with 2.5% and 1.0% CSE respectively. Furthermore cell death after CSE exposure was assessed in A549 and BEAS2B cells stably transfected with PLKO.1 empty vector and shRNA HIF1α construct with or without CSE stimulation. % viable cells in A549 (A) and BEAS2B (C) and total cell count in A549 (B) and BEAS2B (D) were measured. Data are expressed as mean+SD.
(TIF)

Figure S2 **TGF-β induced EMT is prevented by inhibition of the SMAD and MAPK pathway.** Submerged grown A549 cells were treated for 48 h with 2.5% CSE or 10 ng/mL TGF-β. Effect of inhibition of the SMAD pathway was studied using the receptor blocker SB431542 or on the MAPK pathway using the Erk inhibitor U0126 which, both were pre-incubated at a dose of 10 µM for 30 minutes. E-cadherin (A and C) and PAI1 (B and D) mRNA levels were measured by qPCR. Data are expressed as mean+SD, * indicates p<0.05 between CSE stimulated and untreated control.
(TIF)

Figure S3 **A549 cells grown ALI did not show leakage and cell death.** Leakage of A549 cells grown on ALI was measured using the fluorescein leakage test. Leakage of cells on air for 72 h was compared to an empty insert (A). Furthermore cell death over the whole culture period was measured using trypan blue staining (B).
(TIF)
