# Supplementary figures and images for: Cigarette Smoke Extract Induces a Phenotypic Shift in Epithelial Cells; Involvement of HIF1α in Mesenchymal Transition
Source: PLoS One. 2014 Oct 16;9(10):e107757. doi: 10.1371/journal.pone.0107757 (PMC4199572; doi:10.1371/journal.pone.0107757)

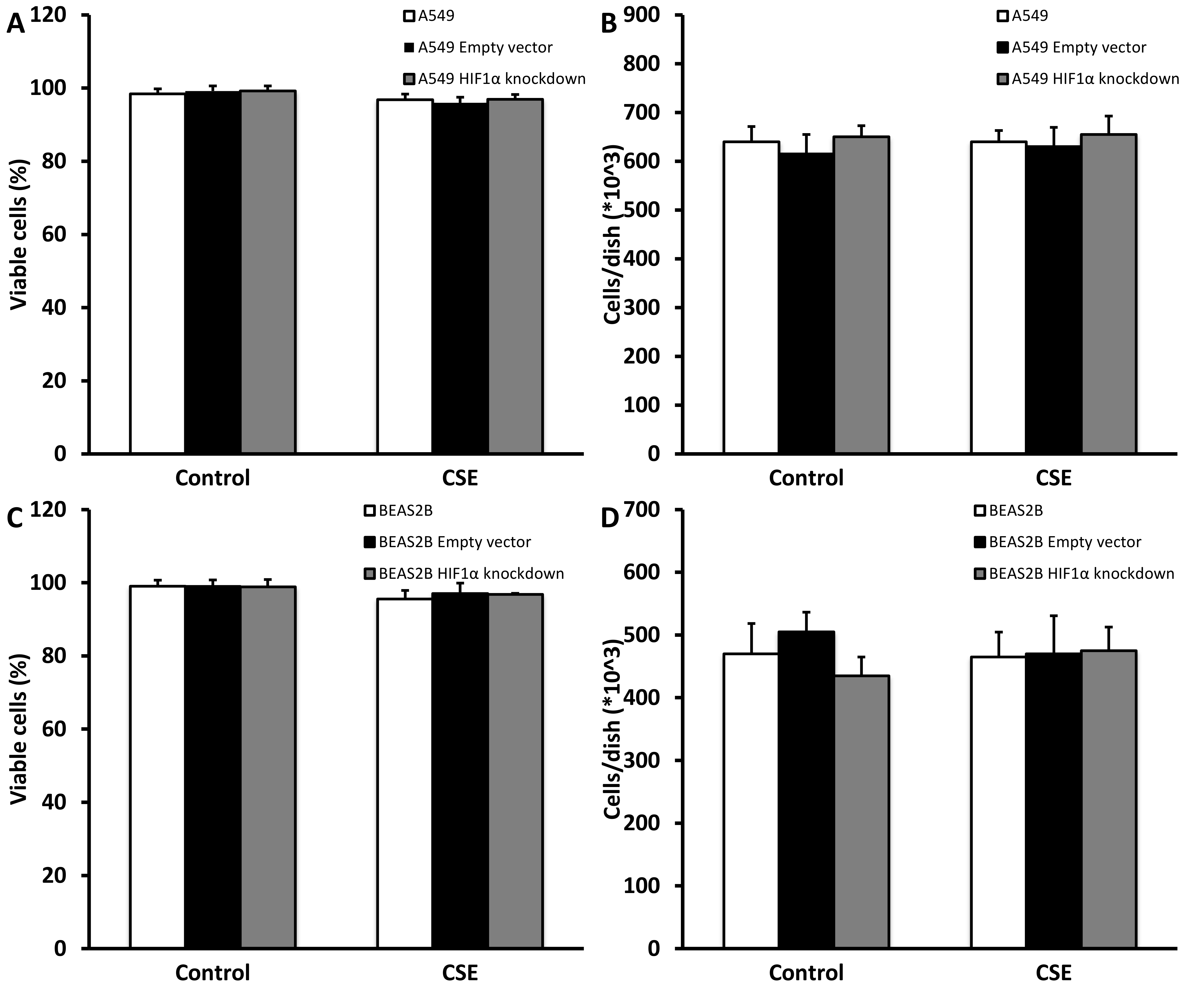

Supplement: Figure S1 — No significant cell death is present after CSE exposure. Cell death was measured using trypan blue staining in A549 and BEAS2B cells after stimulation for 48 h with 2.5% and 1.0% CSE respectively. Furthermore cell death after CSE exposure was assessed in A549 and BEAS2B cells stably transfected with PLKO.1 empty vector and shRNA HIF1α construct with or without CSE stimulation. % viable cells in A549 (A) and BEAS2B (C) and total cell count in A549 (B) and BEAS2B (D) were measured. Data are expressed as mean+SD. (TIF) [file pone.0107757.s001.tif]

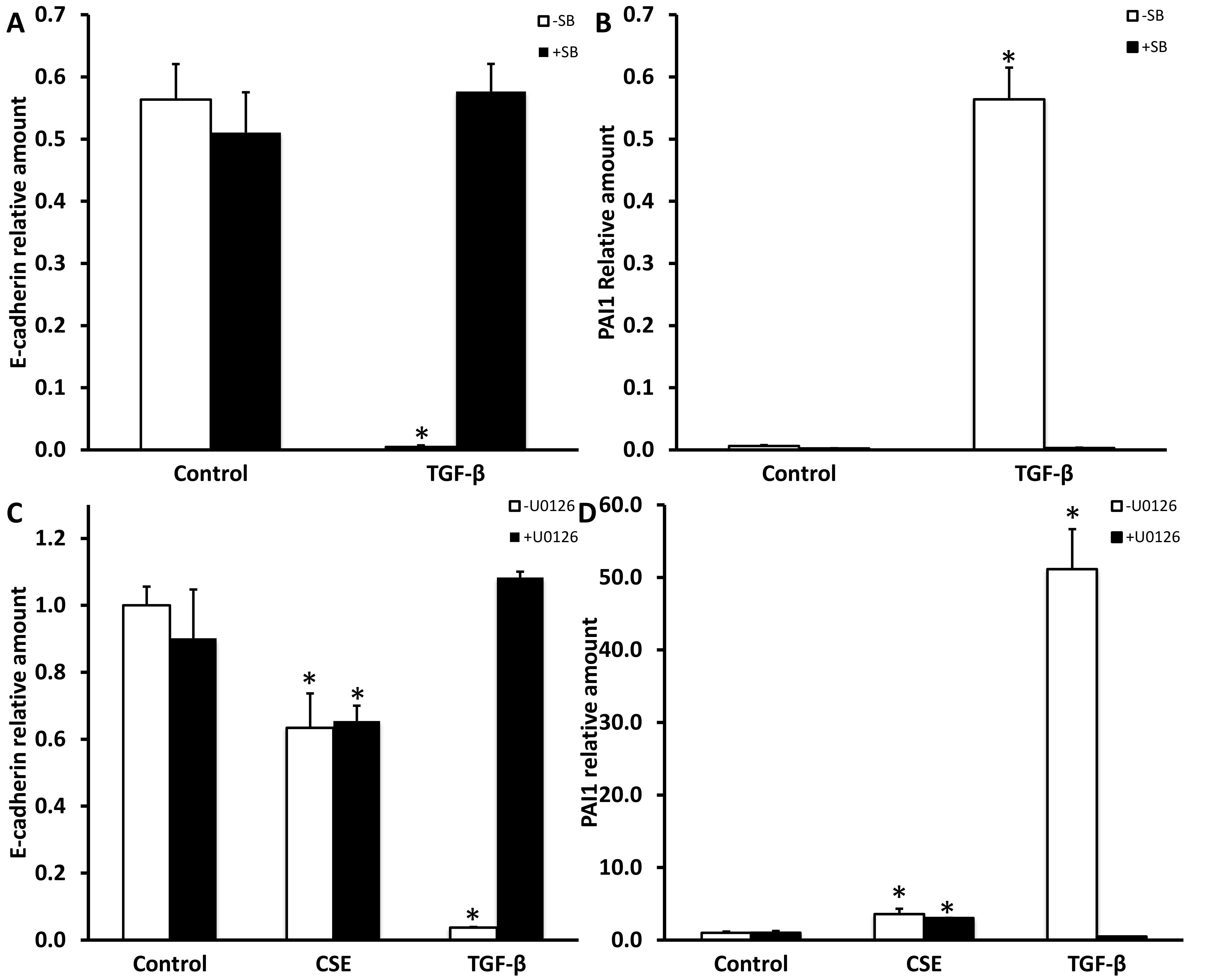

Supplement: Figure S2 — TGF-β induced EMT is prevented by inhibition of the SMAD and MAPK pathway. Submerged grown A549 cells were treated for 48 h with 2.5% CSE or 10 ng/mL TGF-β. Effect of inhibition of the SMAD pathway was studied using the receptor blocker SB431542 or on the MAPK pathway using the Erk inhibitor U0126 which, both were pre-incubated at a dose of 10 µM for 30 minutes. E-cadherin (A and C) and PAI1 (B and D) mRNA levels were measured by qPCR. Data are expressed as mean+SD, * indicates p<0.05 between CSE stimulated and untreated control. (TIF) [file pone.0107757.s002.tif]

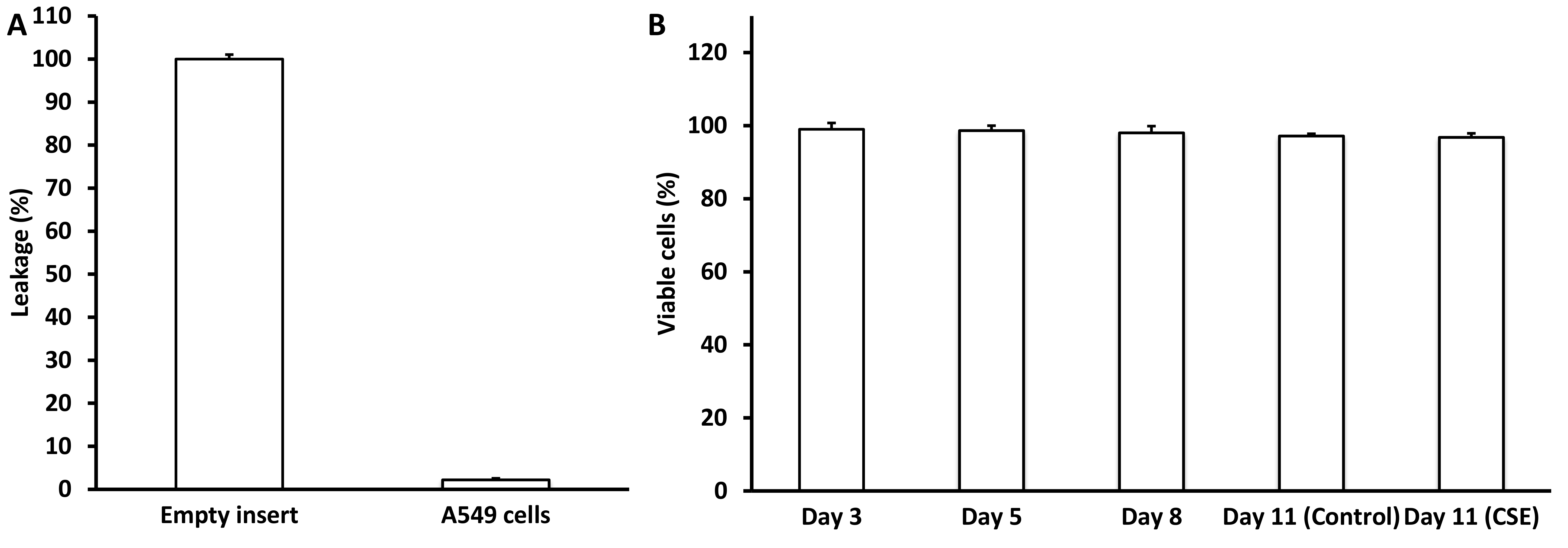

Supplement: Figure S3 — A549 cells grown ALI did not show leakage and cell death. Leakage of A549 cells grown on ALI was measured using the fluorescein leakage test. Leakage of cells on air for 72 h was compared to an empty insert (A). Furthermore cell death over the whole culture period was measured using trypan blue staining (B). (TIF) [file pone.0107757.s003.tif]
